# Applications of Cancer Cell-Specific Aptamers in Targeted Delivery of Anticancer Therapeutic Agents

**DOI:** 10.3390/molecules23040830

**Published:** 2018-04-04

**Authors:** Minhee Kim, Dong-Min Kim, Keun-Sik Kim, Woong Jung, Dong-Eun Kim

**Affiliations:** 1 Department of Bioscience and Biotechnology, Konkuk University, Seoul 05029, Korea; mining9412@gmail.com (M.K.); pie3000@naver.com (D.-M.K.); 2 Department of Biomedical Laboratory Science, Konyang University, Daejeon 35365, Korea; kskim11@konyang.ac.kr; 3 Department of Emergency Medicine Kyung Hee University Hospital at Gangdong, Seoul 05278, Korea; ondali77@khu.ac.kr

**Keywords:** aptamer, targeted delivery, targeted cancer therapy, aptamer conjugates

## Abstract

Aptamers are single-stranded oligonucleotides that specifically bind and interact with their corresponding targets, including proteins and cells, through unique three-dimensional structures. Numerous aptamers have been developed to target cancer biomarkers with high specificity and affinity, and some are employed as versatile guiding ligands for cancer-specific drug delivery and anti-cancer therapeutics. In this review, we list the aptamers that target tumor surface biomarkers and summarize the representative applications of aptamers as agonists and antagonists that activate anti-cancer and inactivate pro-cancer biomarkers, respectively. In addition, we describe applications of aptamer-drug or aptamer-oligonucleotide conjugates that can deliver therapeutic agents, including small interfering RNAs, micro RNAs, short hairpin RNAs, and chemotherapeutic molecules, to cancer cells. Moreover, we provide examples of aptamer- conjugated nano-vehicles, in which cancer-targeting oligonucleotide aptamers are conjugated with nano-vehicles such as liposomes, micelles, polymeric nanoparticles, and quantum dots. Conjugation of aptamers with anti-cancer drugs and nano-vehicles will facilitate innovative applications of aptamer-based cancer therapeutics.

## 1. Introduction

To date, chemotherapy regimens against cancer have serious limitations such as poor tissue selectivity, rapid systemic clearance, low intra-tumoral accumulation, and drug resistance [1,2]. Despite recent revolutionary targeted-therapy, the limitations of chemotherapy are still challenging and problematic [3,4,5]. For these reasons, many patients suffer adverse effects because of myelosuppression and cardiotoxicity before they are relieved of their tumor burdens [6]. To counter these difficulties, targeted delivery of anticancer therapeutic agents directly to cancer cells is a highly desirable strategy to treat malignant tumors without affecting normal cells [7]. To this end, methods for conjugating drug delivery materials to various biomolecules and ligands that are specific to cancer cells have been developed and applied for the targeted delivery of anticancer drugs [8,9,10]. Specifically, surface modifications on nano-sized carriers such as liposomes, micelles, and polymeric nanoparticles with cancer cell-specific antibodies has been used to identify cancer cell-surface targets [11,12,13], which then take up the antibody-conjugated drug delivery vehicles via receptor-mediated endocytosis [14,15]. Although antibody-based drug delivery exhibits selective and improved therapeutic efficacy against several types of cancer cells, protein-antibody conjugations are difficult to control and often have inconsistent binding affinities [16]. In addition, the antibodies used as cell-specific homing agents must be optimized for use in humans to be eligible for clinical application [16,17].

Nucleic acid aptamers are small single-stranded DNA or RNA oligonucleotides (usually 25–90 nucleotide bases) that specifically bind to their target molecules with high affinity and specificity based on a unique three-dimensional structural conformation [18,19,20]. Aptamers typically bind to target molecules with binding affinities in the low nanomolar to picomolar range [21]. Aptamers are created using an in vitro selection process termed systematic evolution of ligands by exponential enrichment (SELEX) [22,23]. As nucleic acid analogs of protein antibodies, an aptamer can specifically bind with high affinity to a broad range of targets, such as small organic molecules, proteins, viruses, or cells [24,25,26,27,28,29]. Thus, nucleic acid aptamers have emerged as an alternative to antibodies for targeted delivery [30]. Nucleic acid aptamers have several advantages over protein antibodies. Aptamers can be obtained in large quantities through chemical synthesis and are much more resistant to heat, pH changes, and organic solvents than antibodies [31]. Aptamers can be denatured and renatured without loss of activity and are thought to be less immunogenic than protein antibodies because they are gradually degraded by nucleases in vivo [32,33,34]. Moreover, aptamers can be chemically modified with diverse functional groups at either the 5′ or the 3′ end to facilitate site-specific conjugation [35]. Nucleic acid aptamers have the advantages of rapid target cell penetration and retention in serum due to excellent stability [36,37]. Thus, DNA or RNA aptamers have been used as tumor-targeting agents instead of protein antibodies in drug-conjugated aptamers, as well as aptamer-conjugated nano-sized vehicles for targeted delivery of drugs to cancer cells.

An important achievement in the application of aptamers to therapeutics is the development of Macugen (also known as pegaptanib), which is used to treat age-related macular degeneration [38,39]. Macugen is an RNA aptamer that antagonizes binding of vascular endothelial growth factor (VEGF) to its receptor. This successful development of a therapeutic aptamer greatly stimulated the exploration of tumor-targeted aptamers for cancer therapy. In addition, various aptamers targeting different cancer markers are currently under preclinical and clinical investigation. One of the most successful tumor-targeted aptamers [40,41], a nucleolin aptamer (AS1411, NucA), is in a phase II trial for the treatment of metastatic renal cell carcinoma and has shown excellent tumor-targeting properties and is non-toxic [42]. A recent search for “aptamer AND drug delivery” in the Medline database showed 639 publications, which accounted for 6% of all publications (10,720) obtained when searching for “targeted drug delivery AND cancer.” This review aims to summarize the application of aptamers as nanomedicines formulated as drug-conjugated aptamers and aptamer-conjugated nano-sized vehicles for targeted cancer therapeutics.

## 2. Development of Cancer Cell-Targeting Aptamers

Aptamers are generally selected from large randomized oligonucleotide libraries by a process known as in vitro SELEX. The SELEX process is a combinatorial biochemical technique to produce oligonucleotides that specifically bind to a target of interest through multiple rounds of selection [43]. This selection process encompasses repeated binding, partitioning, and amplification until the pool of high affinity aptamers is enriched [43]. To prevent exonuclease digestion of nucleic acid aptamers in vivo, several chemical modification methods have been applied to enhance the stability of aptamers. For example, phosphate in the oligonucleotide backbone is replaced with phosphorothioate [44]. In addition, aptamers are readily modified with the addition of poly (ethylene glycol) (PEG) and other moieties to increase the bioavailability and pharmacokinetic properties of oligonucleotide [45]. Endowed with enhanced in vivo stability and biocompatibility, nucleic acid aptamers have emerged as novel and promising molecules that target specific cancer biomarkers for use in diagnosis and therapy. As targeted therapeutics, aptamers can guide drugs to specific cancer lesions by acting as a molecular probe to recognize and bind corresponding receptors.

### 2.1. Development of Aptamers against Biomarkers Using SELEX Technology

SELEX methods can be roughly classified as in vitro, in vivo, and in silico. In the in vitro method, SELEX proceeds through a series of selections for a specific single target of interest (usually purified protein). However, in some circumstances, the selected aptamer may fail to recognize its target on the cell surface [46]. Therefore, in the in vivo (cell-based SELEX) method, the aptamers are isolated using whole cells that express the target protein on their surfaces [27]. Cell-based SELEX follows a similar approach as in vitro SELEX, but live cells are used to express the aptamer selection target. Cell-based SELEX has an advantage over in vitro SELEX, because cell-based SELEX targets native proteins expressed on cell surfaces rather than purified recombinant proteins. Thus, use of cell-based SELEX is more desirable for the selection of aptamers to target cancer cells. Recently, bioinformatics methodologies that combine in vitro and in silico selection have been developed [47,48]. Aptamers are designed with three-dimensional structures that fit the target molecule through a computational approach that reduces the aptamer libraries from 250 million to 10,000 sequences.

A general SELEX process usually consists of three iterative steps. First, the libraries containing approximately 10^15^ unique sequences are incubated with the target of interest [49,50]. During this incubation, only a few sequences bind to the target. In the next step, the sequences tightly bound to the target are separated from those that are unbound or weakly bound to the target sequences. Subsequently, sequences that specifically bind to the target are amplified by PCR. To eliminate non-specific binding of oligonucleotides, in the case of cell-based SELEX, the target cells often must be blocked with tRNA, salmon sperm DNA, or polyinosinic:polycytidylic acid (poly I:C) [51]. Cell-based SELEX is usually performed at 4–37 °C, with aptamers most likely to be internalized into cells by increasing the selection temperature and time during incubation with the randomized oligonucleotide library [52]. PCR amplification of selected aptamers requires optimization to enhance the yield without amplifying non-specific sequences due to mispriming during PCR. Excessive cycles would result in the amplification of non-specific products, whereas fewer cycles may result in insufficient yields [53]. The optimal number of PCR cycles is considered the number of PCR amplification cycles that produce the highest density of PCR amplicon bands without non-specific bands [52]. The PCR product is subsequently used to regenerate the library pool for the next selection cycles, by either RNA transcription or DNA strand separation for RNA aptamers or DNA aptamers, respectively. If DNA aptamers are to be developed, separation of DNA strands is a critical step to regenerate single-strand DNA (ssDNA) libraries. To prepare ssDNA for the next round of SELEX, common methodologies such as asymmetric PCR, electrophoresis-based separation of DNA strands, magnetic beads separation, and lambda exonuclease digestion are often used [23].

After aptamers are selected through the SELEX process, their binding efficiencies against the target molecules must be confirmed. If the aptamers are selected against cancer cells by cell-based SELEX, the aptamers identified should be evaluated using established binding assays such as flow cytometry, fluorescence, or confocal microscopy, or enzyme-linked assay [23]. Aptamers developed by traditional SELEX against specific intra- or extra-cellular markers, which are usually prepared as purified recombinant proteins, are currently more than the number of aptamers selected by cell-based SELEX. Regardless of the aptamer selection process, the binding affinity of aptamers that were developed against a surface cell marker can be evaluated with fluorescence/confocal microscopy and flow cytometry. If the aptamers are developed against intracellular targets, the binding affinity of aptamers can be determined by lysing the cells to release the aptamers for detection [54,55]. Aptamers released from lysed cells can be immobilized on either plates or magnetic beads, which can be detected by enzyme-linked binding assays [54,55,56]. Alternatively, aptamer binding to biomarker proteins can be monitored by changes in light refraction using surface plasmon resonance (SPR).

### 2.2. Aptamers as Cancer Cell-Targeting Agents

Cancer biomarkers are molecules that indicate the abnormal states of cancers and that play important roles in many biological processes, including cell proliferation, cell migration, cell-cell interactions, and signal transduction. Many studies have identified molecules such as membrane proteins, transcription factors, and growth factors as good biomarkers [57,58]. In particular, well-characterized membrane proteins that are endogenously overexpressed on the surfaces of cancer cells are potential targets for cancer therapeutics [59]. Aptamers are sensitive in the detection of these cancer biomarkers and have emerged as novel targeting materials because of their high affinity to target molecules. They recognize and bind to corresponding targets through the formation of spontaneous three-dimensional structures with the aim of improving therapeutic effects and reducing unnecessary toxicity to non-cancerous cells [19]. The major challenge is to identify aptamer sequences that bind to specific biomarkers of cancer cells. In the last few decades, many aptamers specific to tumor-related biomarkers have been developed and extensively studied for therapeutic application to various cancers such as breast cancer [60,61,62], colorectal adenocarcinoma [63], lung cancer [64], and prostate cancer [65], as well as for cancer stem cells [66,67]. Specifically, numerous aptamers that target cancer-specific signature markers such as immunoinhibitory programmed death-1 [68], immune stimulating CD137 [69], CD134 [70], tumorigenic platelet-derived growth factor [71], and vascular endothelial growth factor [72] have been established. We summarize currently known cancer biomarkers and their respective aptamers in Table 1.

A clinically renowned aptamer in anti-cancer research is AS1411, a 26-nucleotide guanosine-rich DNA sequence that specifically binds to overexpressed or translocated nucleolin (NCL) in many types of cancer cells [73]. In addition to its cancer-targeting efficacy, AS1411 blocks binding of NCL to the Bcl-2 oncogene, thereby inhibiting cell escape from apoptosis [64,74]. Several preclinical studies of AS1411-conjugated nano-vehicles at very low concentrations have shown significant inhibitory effects on various tumor cell lines with minimal side effects [75]. Inspired by the preclinical success of AS1411, its commercial version (generated by Aptamera Inc., Louisville, KY) is under phase II clinical trials for its anti-acute myeloid leukemia (AML) and renal cell carcinoma (RC) [76] effects. Another well-known aptamer with potential clinical application is A10, which specifically binds to a prostate cancer biomarker, prostate-specific membrane antigen (PSMA) [77]. Many studies have demonstrated significant effects such as specific in vivo therapeutic efficacy against prostate cancer in a PSMA-expressing LNCaP cell xenograft mouse model [78,79,80,81].

## 3. Aptamer-Mediated Therapeutics against Cancer

Aptamer-mediated targeted therapeutics generally employs one of three strategies. (1) Aptamers can act as antagonists or agonists to inhibit or stimulate, respectively, the interactions of tumor-associated targets; (2) Aptamers can be covalently or non-covalently conjugated with drugs to form aptamer-drug conjugates (ApDCs). For example, doxorubicin (Dox) is effectively loaded onto aptamers by intercalation at specific paired GC sites in the aptamer sequence. Furthermore, aptamers can serve as carriers to deliver therapeutic molecules to cancer cells; (3) Aptamers can be applied to novel nanoparticles to increase the therapeutic response. Aptamer-conjugated nano-vehicles carrying anti-cancer drugs exert tumoricidal therapeutic effects, in which the aptamers guide the therapeutic reagents to the extracellular region of a tumor-specific surface biomarker.

### 3.1. Aptamers as Cancer Cell Agonists and Antagonists

**Figure 1:**
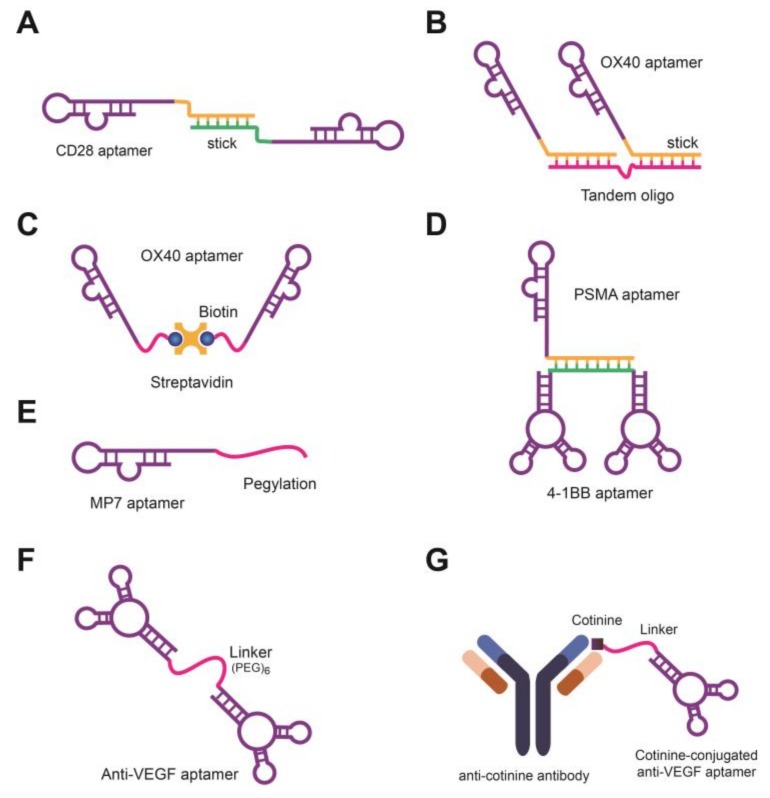
Schematics of aptamers used as agonists or antagonists against cancer biomarkers. (**A**) Bivalent CD28 aptamer conjugate. CD28 aptamers are linked through 21 base-paired double-stranded RNA molecules; (**B**) Bivalent OX40 aptamer conjugate. The 3′-end stick sequence of OX40 aptamers are annealed to a DNA scaffold; (**C**) Bivalent OX40 aptamer conjugate. Biotin modified OX40 RNA aptamers are assembled via streptavidin; (**D**) Bispecific PSMA-4-4BB aptamer conjugate. A PSMA aptamer and bivalent 4-1BB aptamer are non-covalently annealed with a stick sequence; (**E**) An MP7 aptamer is conjugated with PEG; (**F**) Bivalent anti-VEGF aptamer. Two aptamers are tethered through a hexaethylene glycol spacer; (**G**) Anti-VEGF aptamer-antibody conjugate. This “oligobody” was developed to improve in vivo therapeutic responses.

Because aptamers show a remarkable affinity and specificity in targeting ligands, they can be used to stimulate or inhibit a target of interest, such as the receptors and growth factors responsible for cancer progression [93]. For example, RNA aptamers against murine CD28 have been developed and used as antagonists or agonists, depending on their form [94]. The monomeric aptamer CD28Apt2 acts as an antagonist that inhibits interactions between CD28 and the B7.2 ligand, reducing immunogenic signals. In contrast, bivalent aptamers that are linked by 21-base paired double-stranded RNA function as agonists (Figure 1A). The agonistic aptamers co-stimulate CD8 T cells and CD4 lymphocytes and promote cellular immune responses, resulting in the survival of mice. The agonistic OX40 that targets CD134 and 4-1BB that targets CD137 were shown to enhance anti-tumor responses through T cell activation [69,70]. In addition, two OX40 aptamers annealed on a scaffold DNA (tandem oligo) and the resulting bivalent aptamer were shown to activate primed T cells both in vitro and in vivo (Figure 1B). Recently, a biotin-streptavidin-conjugated bivalent OX40 RNA aptamer was developed and found to promote T cell proliferation and interferon production (Figure 1C) [95]. The bi-specific PSMA-4-1BB aptamer conjugate consists of a PSMA aptamer and bivalent 4-1BB aptamer (Figure 1D) [69]. This combination of tumor targeting aptamer (PSMA aptamer) and immunogenic aptamer (bivalent 4-1-BB aptamer) was shown to reduce side effects and improve therapeutic responses.

PD-1, which suppresses the inflammatory activity of T cells by binding to PD-L1, is a novel target for cancer therapies [96]. The MP7 aptamer specifically binds to the extracellular region of the PD-1 receptor on T cells and antagonizes PD-1-mediated immune-suppression [68]. Furthermore, the MP7 aptamer conjugated with polyethylene glycol, which extends the half-life of the aptamer up to 24–48 h, suppresses tumor growth without triggering TLR9-mediated innate immune signals (Figure 1E). The anti-PDGF RNA aptamer (ARC126) and anti-VEGF aptamer (pegaptanib) are antagonistic aptamers that inhibit angiogenesis in various cancers. The former has been tested in phase II clinical trials, and the latter has been commercialized [39,97,98]. To increase their biocompatibility, two anti-VEGF aptamers were tethered to a hexaethylene glycol spacer (Figure 1F). Recently, an aptamer-antibody conjugate called an “oligobody” (oligomer + antibody) has also been developed for improved in vivo anti-cancer efficacy (Figure 1G) [99].

### 3.2. Aptamer-Drug Conjugates for Targeted Drug Delivery

**Figure 2:**
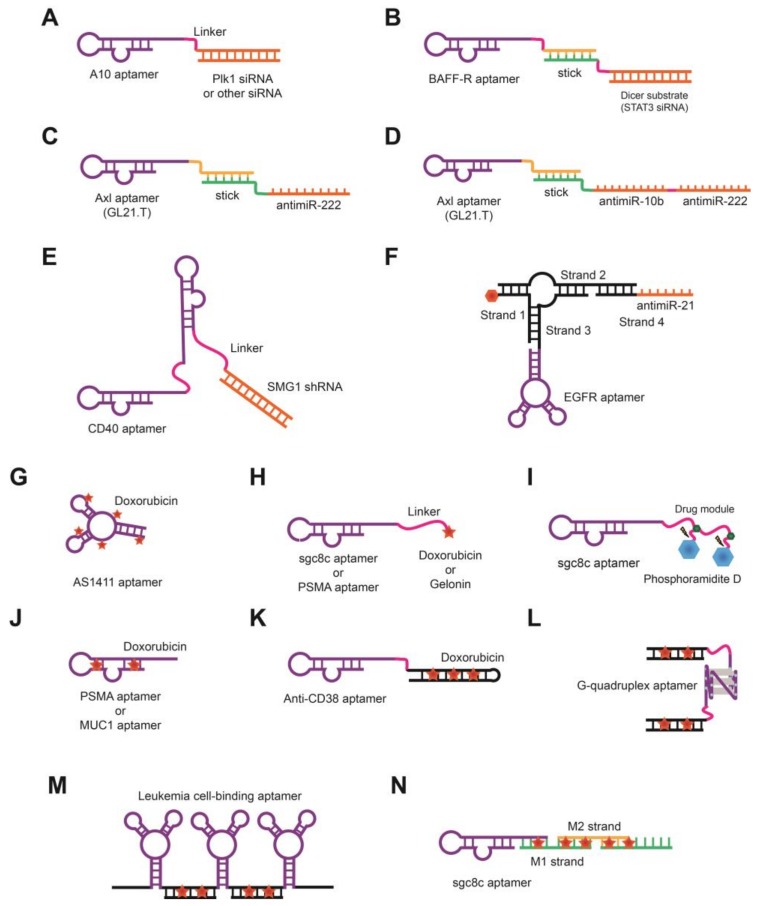
Schematics of aptamer-oligonucleotides and aptamer-drug conjugates. (**A**) Aptamer-siRNA conjugate. A PSMA aptamer is covalently conjugated with Plk1 siRNA via a linker; (**B**) Aptamer stick sequence-siRNA conjugate. A BAFF-R aptamer is complexed with STAT3 siRNA via a pair of complementary stick sequences; (**C**) Aptamer stick sequence-miRNA conjugate. The stick sequence (17-mer) of the GL21.T aptamer and extended stick sequence of anti-miRNA-222 at the 3′-end are fully annealed; (**D**) Aptamer stick sequence-dual anti-miRNA conjugate. Anti-miRNA-10b and anti-miRNA-222 are conjugated with the GL21.T aptamer; (**E**) Bivalent aptamer-shRNA conjugate. Two CD40 aptamers are conjugated with SMG1 shRNA; (**F**) Three-way junction-aptamer-anti-miRNA conjugate. The trifunctional aptamer consists of four DNA strands containing EGFR aptamer, anti-miRNA-21, and Alexa 647 dye; (**G**) Aptamer-drug conjugate. An AS1411 aptamer is directly conjugated with Dox by incubation with formaldehyde; (**H**) Aptamer-linker-drug conjugate. An Sgc8c aptamer and Dox or PSMA aptamer and gelonin are conjugated via a linker; (**I**) Aptamer-multiple drug conjugate. Sgc8 aptamer is conjugated with modified phosphoramidite containing a photocleavable linker and an anti-cancer drug; (**J**) Drug-intercalating aptamer. Dox is loaded into double-stranded regions of the PSMA aptamer or MUC1 aptamer; (**K**) Aptamer-cargo conjugate. A CD38 aptamer conjugated with the CG-rich sequence of “CG cargo” carries Dox; (**L**) Aptamer-dual cargo conjugate. A G-quadruplex DNA aptamer is conjugated with two double-stranded DNAs carrying Dox by a linker; (**M**) Polyvalent aptamer-drug conjugate. Multiple aptamers specific to leukemia cells are synthesized by rolling circle amplification and Dox is loaded between the aptamer units; (**N**) Aptamer-tethered DNA nanotrains. An Sgc8c aptamer and two short DNA strands were assembled to form “nanotrains”.

In addition to therapies that use aptamers alone, aptamers can be used to specifically deliver other therapeutic agents, including small interfering RNAs (siRNAs), micro RNAs (miRNAs), short hairpins RNA (shRNA), and chemotherapeutics. A variety of aptamers specific to cancer biomarkers have been used to deliver therapeutic agents with increased local concentrations and treatment efficacy. Aptamers can be covalently or non-covalently conjugated with drugs and therapeutic oligonucleotides to form aptamer-drug conjugates (ApDCs) and aptamer-therapeutic oligonucleotide conjugate (ApOCs), respectively. The simplest linkage for aptamer-therapeutic oligonucleotide conjugates is through covalent conjugation. A PSMA aptamer-siRNA conjugate targeting prostate cancer was developed to suppress the expression of pro-survival genes such as polo-like kinase 1 (*PLK1*) and B cell lymphoma 2 (*BCL2*) (Figure 2A) [100].

Aptamers can also be non-covalently conjugated through extra-extended strands called “sticky bridges.” For example, the B cell-activating factor receptor (BAFF-R) aptamer that can bind to BAFF-R was selected to suppress BAFF-R-mediated cancer [101]. The BAFF-R aptamer was complexed with STAT3 siRNA, which plays an important role in B cell lymphoma progression, via two complementary stick sequences (Figure 2B). Using the same strategy, the GL21.T aptamer targeting the receptor tyrosine kinase Axl was conjugated with anti-miRNA-222 (Figure 2C) [102]. The 17-mer at the 5′-end of the aptamer and anti-miRNA-222, extended at the 3′-end, are fully complementary to each other. The resulting aptamer-anti-miRNA conjugate reduced miRNA-222 levels in Axl-expressing cells. To increase the therapeutic effect by antagonizing corresponding onco-miRNAs independently, anti-miRNA-10b and anti-miRNA-222 were both joined with aptamer GL21.T (Figure 2D). The GL21.T aptamer was non-covalently conjugated with bi-modular anti-miRNA-10b and anti-miRNA-222, which antagonized the miRNAs in vitro and in vivo. Dually functional CD40 aptamers that consist of two parts, including shRNA with different functions, have also been designed (Figure 2E) [103]. The agonistic bivalent CD44 RNA aptamer binds and activates B lymphocytes to ameliorate bone marrow aplasia. In addition, shRNA against SMG1 was employed to inhibit nonsense-mediated mRNA decay. The bivalent CD40 aptamer-SMG1 shRNA chimera was reported to improve immune responses and consequently overall survival in vivo. In addition to therapeutics, ApOC has also been used as an imaging tool. A trifunctional aptamer has been developed that consists of an epidermal growth factor receptor (EGFR) aptamer as a targeting molecule, anti-miRNA-21 as a therapeutic agent, and Alexa 647 dye as an imaging agent into an RNA scaffold in a three-way junction (Figure 2F) [104]. This trifunctional aptamer was reported to decrease miRNA-21 levels and inhibit tumor growth in a breast cancer-bearing mouse model, while allowing imaging to track cancer cells.

Aptamers can be chemically synthesized and easily modified. In this regard, therapeutic agents, including chemotherapeutic molecules (i.e., Dox), toxins, and proteins, can be easily conjugated to aptamers. These conjugates have been shown to reduce side effects and enhance efficacy with liver cancer cell-specific drug delivery in tumor-bearing mice model. The anti-cancer drug Dox was covalently conjugated with the AS1411 DNA aptamer, which targets plasma membrane nucleolin, a protein that is overexpressed in many cancer cell types [105]. The AS1411 aptamer-Dox conjugates were prepared by incubating the aptamer and Dox with a crosslinking agent, formaldehyde (Figure 2G). In another example, Sgc8c aptamer-Dox conjugates were specifically delivered to protein tyrosine kinase 7 (PTK7)-expressing cells and were cytotoxic to the target leukemic cells (Figure 2H) [106]. In the same way, the PSMA aptamer was conjugated with ribosomal toxin gelonin, which cleaves 28S ribosomal RNA at nucleotide 4324 [107]. In addition, the Sgc8 aptamer was synthesized with derivatization, using a modified phosphoramidite containing a photocleavable linker and anti-cancer drug (Figure 2I) [108]. The chemically synthesized aptamer-drug conjugates targeted the expected cancer cells, with the release of drugs regulated by UV irradiation.

Alternatively, Dox can be non-covalently intercalated into aptamer sequences, as Dox has a high affinity to GC-rich double-stranded regions of DNA because of its planar ring structure. Thus, drug-intercalated aptamers can act as both targeting ligands and drug carriers [109]. Dox was initially loaded into double-stranded regions of the PSMA aptamer (Figure 2J) [110], with Dox and PSMA aptamers incubated at about 1:1.2 molar equivalence. The Dox-loaded PSMA aptamer showed remarkable specificity and cellular uptake in PSMA-positive cells, but not in PSMA-negative cells. Dox was also intercalated into DNA aptamer MA3 that targeted MUC1, which is overexpressed on the surfaces of various cancer cells (Figure 2J) [111]. The MA3-Dox complex showed specific binding and cellular uptake of Dox with significant cytotoxicity in MUC1-positive tumor cells, but not in MUC1-negative cells. CD38 DNA aptamers harboring CG-rich regions are called “CG cargo.” They can carry a high payload of Dox in their CG-repeat structures and have been loaded with Dox for drug delivery to CD38-positive multiple myeloma (MM) cells (Figure 2K) [112]. In the low pH environment inside of cells, Dox was rapidly released following structural changes in the aptamer carrier. In addition, when CD38 DNA aptamers loaded with Dox were administered systematically to MM tumor-bearing mice, a specific internalization of Dox into tumor cells, suppressed tumor growth, and increased survival was observed in the mice. A double-stranded DNA aptamer (ApS) nanoparticle was established to deliver the Dox to the cancer cells, with the ApS nanoparticles composed of G-quadruplex DNA aptamers (Figure 2L) [113]. These aptamers target nucleolin, expressed on cancer cells, and their double-stranded DNA contains CG-rich regions for Dox intercalation. The intercalation of Dox into the double-stranded DNA had no effect on the G-quadruplex structure of the aptamer. In addition, a poly-aptamer-drug system was developed (Figure 2M) that contains multiple aptamers synthesized by rolling circle amplification and Dox intercalated between aptamer units [114]. The poly-aptamer-drug complex showed increased binding affinity (40-fold greater) to target cells, compared to that of the aptamer alone. This study demonstrated higher affinity and cytotoxicity of multiple aptamers at close range than those of monovalent forms. Further, aptamer-tethered DNA have been shown to self-assemble, with two short DNA strands named “nanotrains” (aptNTrs) used for efficient Dox loading (Figure 2N) [115]. The aptNTrs transported Dox to cancer cells and selectively induced cytotoxicity with antitumor efficacy in a xenograft mouse.

### 3.3. Aptamer-Conjugated Nano-Vehicles for Targeted Drug Delivery

Although Dox-intercalated aptamers decreased off-target cytotoxicity, reducing side effects relative to that of the free drug, Dox intercalation into DNA duplexes may cause major DNA structural changes, potentially reducing the specificity of aptamers. Agudelo et al. examined DNA conformational changes induced by Dox intercalation [116]. The NH_2_ group on Dox was critical for intercalation, participating in a structural transition from a partial B to A-DNA form. Furthermore, Park et al. compared the efficiency of Dox delivery by Dox-intercalated aptamers and Dox-encapsulating aptamer-conjugated liposomes [117]. Dox-intercalated aptamers were less effective than Dox-encapsulating aptamer-conjugated liposomes for drug delivery and targeted cytotoxicity. These results suggested that maintaining an intact aptamer structure is necessary for aptamers to be used as targeting molecules.

**Figure 3:**
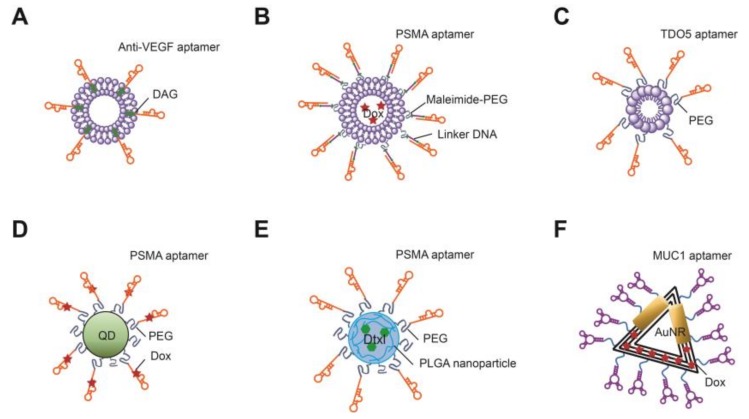
Schematics of aptamer nano-vehicle conjugates. (**A**) Aptamer-conjugated liposome. NX213 aptamers are directly conjugated with liposomes; (**B**) An aptamosome. A PSMA aptamer is annealed to linker DNA and conjugated with liposomes to form Dox-encapsulating “aptamosomes”; (**C**) Aptamer-micelle conjugate. A TDO5 aptamer is conjugated with a PEG-modified lipid to form aptamer-functionalized micelles; (**D**) Aptamer-nanoparticle conjugate. A Dox-encapsulated PSMA aptamers is conjugated with a quantum dot; (**E**) Aptamer-polymeric nanoparticle conjugate. An aptamer is conjugated with a PLGA nanoparticle containing docetaxel; (**F**) Aptamer-DNA origami conjugate. MUC1 aptamers are conjugated with DNA origami harboring gold nanorods and Dox.

To overcome this problematic distortion in aptamer structure, aptamers can be conjugated with nano-vehicles such as liposomes, micelles, polymeric nanoparticles, and quantum dots (QDs), which preserves the aptamer structure. For example, anti-VEGF aptamer (NX213) has been conjugated with liposomes (Figure 3A) [90]. The aptamer-conjugated liposomes reduced angiogenesis in a chicken chorioallantoic membrane model in vivo. Liposomes can carry highly toxic drugs, and their surfaces can be easily modified with polyethylene glycol (PEG) to reduce non-specific lipid fusion with membranes. Beak et al. reported a PSMA RNA aptamer-conjugated liposome called an “aptamosome” that can encapsulate Dox (Figure 3B) [81]. This PSMA aptamosome specifically binds to PSMA-positive prostate cancer cells, but not to PSMA-negative cells, and shows selective toxicity for PSMA-positive cells. In an LNCaP cell xenograft mice model, the biodistribution of and tumor growth inhibition by this aptamosome with a therapeutic potential were demonstrated in vivo. For better penetration and increased binding affinity, a simple TDO5 DNA aptamer targeting the immunoglobulin heavy chain receptor was conjugated to lipid tails via PEG to formulate an aptamer-micelle complex (Figure 3C) [118]. The abilities of the aptamer and aptamer-micelle to bind to target cells were compared, and the aptamer-micelle showed more rapid and sensitive targeting.

Several studies have examined gold nanoparticles (AuNPs) as aptamer-conjugated carriers. AuNPs can be easily conjugated with thiol group (-SH)-modified aptamers, and these conjugates show low toxicity and high biocompatibility. As an example, a PDGF aptamer was covalently conjugated to AuNPs [119]. The resulting Apt-AuNPs specifically suppressed the proliferation of MDA-MB-231 breast cancer cells. In addition, a QD was conjugated with a Dox-intercalating PSMA aptamer, enabling simultaneous cancer imaging and therapy (Figure 3D) [120]. The conjugates initially showed no fluorescence in a bi-fluorescence resonance energy transfer (FRET) system, because the green fluorescence of the QD was quenched by Dox, and the red fluorescence of Dox was quenched by the duplex RNA aptamer. When the conjugates were taken up by PSMA-positive cells, Dox was released from the aptamer, and the QD and Dox fluorescence were subsequently activated.

Poly lactic-co-glycolic acid (PLGA) is a copolymer synthesized from two different monomers, the cyclic dimers of glycolic acid and lactic acid via ring-opening co-polymerization. Well-controlled PLGA polymerization can produce nanoparticles and PLGA is used for targeted therapies with drugs using aptamers as targeting molecules (Figure 3E). Three different research groups have developed PSMA aptamer-PLGA-PEG nanoparticles harboring docetaxel [79], MUC1 aptamer-PLGA-PEG nanoparticles harboring paclitaxel [121], and CD133 aptamer-PLGA-PEG nanoparticles harboring propranolol [122]. All of these aptamer-PLGA-PEG nanoparticles with drugs showed sustained release of their drugs and higher cytotoxicity for target cells than the free drugs. Interestingly, the number of aptamers on the nanoparticles can be controlled by changing the mixing ratio of the PSMA aptamer-PLGA-PEG and PLGA-PEG copolymer. The optimal number of PSMA aptamers was determined to range from 10 to 80 nmol per μmol of nanoparticle for cancer treatment. Zhu et al., used repeated DNA segments generated by rolling circle amplification to create self-assembled DNA “nanoflowers” (NFs) [123]. The repeating sequences contain Sgc8 aptamer sequences for targeting and CG-rich sequences for Dox intercalation. Moreover, the aptamer-NFs were used as cancer imaging agents by incorporating a fluorophore. In a similar way, four single-stranded DNAs were self-assembled to form an aptamer-based DNA nano-assembly (AptNA) [124]. The multifunctional DNA strands, including Sgc8 aptamers and acrydite groups, were assembled to form a Y-shaped domain and annealed with an X-shaped connector domain. The building units of the nano-assembly consisted of three Y-shaped domains and one X-shaped domain. An acrydite-mediated photo cross-reaction increased the linkages to form AptNA. Dox-loaded AptNA was delivered much more efficiently than free Dox, resulting in higher cytotoxicity to the target cancer cells than off-target cells.

DNA origami, which is a nanostructure created by folding a long stretch of single-stranded DNA with short staple DNAs, has also been developed as a novel drug delivery agent [125]. Single-stranded M13mp18 DNA, hundreds of staple strands, and aflatoxin B1 (AFB1) aptamer-conjugated staple strands were constructed to form functional aptamer-DNA origami conjugates. Dox and gold nanorods (AuNR), which are photothermal therapeutic agents, were added to the aptamer-DNA origami conjugates (Figure 3F) [126]. Target cancer cells were effectively treated with MUC1 aptamer origami-Dox-AuNR (MDOA) because of the synergistic effect of chemo-thermal therapy.

Despite hundreds of preclinical studies that confirmed the therapeutic effect of aptamer-based drug delivery, many of them showed limited effects in clinical evaluations [42]. One of the major reasons for this is tumor heterogeneity [127,128]. Tumor heterogeneity is acquired by gene mutations during replication, adaptation in various tissue environments, survival of a subset of tumor cells after treatment, and recovery from various metastatic states [129,130,131]. Tumor heterogeneity makes targeted therapy difficult, for example, by reducing the target specificity of aptamers or allowing the tumor cells remaining after treatment not to express the target biomarker. One possible solution is a dual-aptamer strategy, which combines two aptamers against major biomarkers for certain cancers. In one example, PSMA aptamers for PSMA-positive prostate cancer cells and DUP-1 peptide aptamers for PSMA-negative were conjugated with thermally cross-linked superparamagnetic iron oxide nanoparticles (TCL-SPION) [132]. In vivo evaluation indicated that the Dox-loaded aptamer TCL-SPION was selectively taken up by both cells but not by normal cells (HeLa cell). MUC1 aptamers and HER2 aptamers were also conjugated with silica nanoparticles for targeted treatment of circulating tumor cells in breast cancer [133]. Taghdisi et al. even developed a DNA dendrimer containing three different aptamers [134]; three respective aptamers targeting MUC1, nucleolin, and ATP were conjugated to nanoparticles harboring epirubicin.

Aptamers have been also used to target cancer stem cells (CSCs), which are a subset of cancer cells that have stem-like properties, including self-renewal and differentiation abilities. CSCs are believed to be a major driving force for tumor heterogeneity and metastatic relapse [135]. Several researchers have reported CSC-targeted therapies based on aptamers against CSC biomarkers. Aptamers for EpCAM, a well-known marker for colon CSCs were functionalized to form PLGA-lecithin-curcumin-PEG nanoparticles (Apt-CUR-NPs) [84]. The Apt-CUR-NPs effectively delivered curcumin to EpCAM-expressing cells and showed greater cytotoxicity than free curcumin. Aptamers specific to the CD44 receptor, which is highly expressed in many types of CSCs, were conjugated with Dox-encapsulating liposome (Apt-Lip) [67]. The binding affinity of this conjugate to CD44 antigen was improved by multivalent binding of Apt-Lip, compared to that of the aptamer alone; consequently, Apt-Lip delivered more Dox to the target CD44+ cancer cells.

## 4. Conclusions

Nucleic acid aptamers are reliable substitutes for conventional antibodies with several advantages. Oligonucleotide aptamers are easily isolated through SELEX and can be chemically synthesized with great accuracy and consistent quality. In addition, they are highly stable under experimental or bioenvironmental conditions, and can be chemically modified with diverse molecules. Moreover, aptamers are small enough to penetrate deep into tumors, and they show few immunogenic effects. Because of these advantages, the use of aptamers is a promising strategy to overcome existing problems of common anticancer therapies. In this review, we focused on aptamers specific to cancer biomarkers, which not only indicate abnormal states of cells, but also play important roles in cancer progression. As cancer-targeting therapeutic agents, aptamers can be agonists that activate the function of targets related to cancer suppression or immunity, such as CD28, CD134, and CD137. Alternatively, aptamers can serve as antagonists, inhibiting targets related to cancer progression, such as PD-1, PDGF, and VEGF. In addition, aptamers can act as both drugs and targeting agent, and oligonucleotide aptamers can deliver various therapeutic agents, including small siRNAs, miRNAs, shRNAs, chemotherapeutic molecules, and many nanoparticles. Aptamer-drug or aptamer-oligonucleotide conjugates are an efficient means of delivering therapeutic substances to targeted cancer cells. For effective anti-cancer drug delivery, aptamers themselves can be used as drug carriers; Dox easily intercalates into CG-rich sequences of aptamers. The resulting Dox-intercalated aptamers show high cytotoxicity and specificity for cancer cells. However, Dox induces conformational changes in DNA during intercalation and therefore may reduce the affinity or specificity of aptamers for targets. To deliver drugs while preserving the aptamer structure, cancer-targeting oligonucleotide aptamers have been conjugated with nano-vehicles such as liposomes, micelles, polymeric nanoparticles, and QDs. Using different anti-cancer drugs with diverse aptamer-conjugated nano-vehicles, ranging from gold nanoparticles to DNA origami, cancer cell-specific aptamers have been widely applied for targeted delivery of anticancer therapeutic agents and have shown remarkably specific cytotoxicity. Applications of aptamer-based targeted anticancer therapy as presented in figures were summarized with their functions in Table 2.

## Figures and Tables

**Table 1:** Aptamers targeting cancer-related biomarkers.

Target	Known Expressing Cancer Type	Therapeutic Applications	Aptamer/Type (nts)	Reference
MUC1 (mucin 1)	ovarian, breast, lung, pancreatic cancers, multiple myeloma etc.	Prevent cancer cell invasion through beta catenin	S2.1/DNA (25)	[60]
			Apt/DNA (25)	[82]
HER2 (human epidermal growth factor 2)	Breast, gastric, lung, colorectal, esophageal, ovarian cancers, etc.	Inhibition of tumorigenic signaling via MAPK, PI3K, PKC and STAT pathways	HB5/DNA (86)	[61]
			Apt/DNA (31)	[83]
HER3 (human epidermal growth factor receptor 3)	Breast, lung, gastric, prostate, ovarian, pancreatic cancers etc.	Reduction of drug resistance in HER2+ cancer	A30/RNA (49)	[62]
EpCAM (epithelial cell adhesion molecule)	Bladder, breast, colon, lung, ovarian, pancreas, prostate cancers, etc.	Regulate gene expression of c-myc, e-fabp, cyclin, and modulate EMT	SYL3/DNA (80)	[63]
			Apt/RNA (18)	[84]
			EpDT3-DY647/RNA (19)	[66]
NF-kB (nuclear factor kappa-light-chain-enhancer of activated B cells)	Cervical, prostate, lung, breast cancers, etc.	Inhibit the genes that control cell proliferation and cell survival	Apt/RNA (29)	[85]
			ARGO100/DNA (26)	[64]
PSMA (prostate specific membrane antigen)	Prostate, kidney, bladder cancers, etc.	Prevent hydrolysis of N-acetylaspartyl-glutamate for over-proliferation	xPSM-A10/RNA (40)	[64,78]
			Apt/DNA (32)	[86,87]
CD44	Breast, prostate, and cancer stem cells, etc.	Inhibit cell proliferation, differentiation, migration, and angiogenesis	TA1/DNA (30)	[67,88]
PD-1 (programmed death-1)	Colon cancer, carcinoma, etc.	Inhibiting immune response to cancer cells	MP5, MP7/DNA (75)	[68]
CD137 (4-1BB)	Prostate cancer etc.	Stimulating immune response to cancer	PSMA-4-1BB/RNA (293)	[69]
CD134 (OX40)	Melanoma tumor etc.	Stimulating immune response to cancer	Aptamer 9.8/RNA (80)	[70]
PDGF (platelet derived growth factor)	Ovarian, breast, thyroid, cervical, lung cancers, etc.	Inhibit tumor angiogenesis and development	36t/DNA (39)	[71,89]
VEGF (vascular endothelial growth factor	Breast, brain, lung, colon, gastric, pancreatic, melanoma, myeloid, leukemia, etc.	Prevent neovascularization	NX-191/RNA (24)	[72]
			NX-213/RNA (24)	[90]
			Vap7, V7t1/DNA (25)	[91,92]
NCL (Nucleolin)	Leukemia, gastric, breast cancers etc.	Induce *bcl-2* mRNA instability	AS1411/DNA (26)	[74]

**Table 2:** Summary of aptamer applications and their functions in cancer therapy.

Type	Name of Aptamer Drugs	Function in Cancer Therapy	Reference
Agonist & Antagonist	CD28Apt	Either reducing the T-cell tolerance by blocking the interaction with B7 or enhancing the vaccine-induced immune response	[94]
	OX40 aptamer	Stimulating the T cell proliferation and cytokine production	[70,95]
	PSMA-4-1BB aptamer	Promoting the survival and expansion of activated CD8+ T cells	[69]
	PEG-MP7	Inhibiting the PD-L1-mediated suppression of IL-2 secretion in T cells	[68]
	NX1838 aptamer	Binding to VEGF_165_ with high affinity and preventing blood vessel growth and arresting the progression	[98]
	Cot-pega oligobody	Inhibiting the Akt pathway that induces the cell survival, angiogenesis, differentiation, cell growth, proliferation	[99]
Aptamer-drug conjugates	A10-Plk1	Suppressing the expression of polo-like kinase 1 that pro-survival genes	[100]
	BAFF-R-STAT3 siRNA	Blocking the BAFF-mediated proliferation of B-cell malignancies and suppressing the transcription factor STAT3 to inhibit the cell cycle progression, angiogenesis and tumor cell evasion of immune system	[101]
	GL21.T-222	Inhibiting the receptor tyrosine kinases Axl and PDGFR β and reducing the level of miR-222 or miR-10b	[102]
	CD40-SMG1-shRNA chimera	Inhibiting SMG1 kinase that is essential for nonsense mRNA mediated decay initiation in tumor cells	[103]
	3WJ-EGFRapt/anti-miR-21	Inhibiting of tumor progression, invasion, and metastasis by suppressing of miR-21	[104]
	AS1411-Dox	Inhibiting of tumor cell proliferation by inducing G2/M arrest	[105]
	Sgc8c-Dox	Recognizing the protein tyrosine kinase 7 and delivering Dox to the target CCRF-CEM (T-cell Acute Lymphoblastic Leukemia) cells	[106]
	ApDCs	Recognizing target cancer cells and release the Fluorouracil in a photocontrollable manner	[108]
	MA3 Apt-Dox	Selectively delivering the cytotoxic agent doxorubicin to MUC1-positivie adenocarcinomas cancer cells	[111]
	ApDC	Delivering the Dox to CD38-positive m1ultiple myeloma tumor cells and intracellular release of a high drug payload under a pH-controlled mechanism	[112]
	ApS&Dox	Targeting nucleolin molecule and circumventing Dox resistance by cell cycle arrest in S phase, effectively increased cell uptake	[113]
	Poly-Aptamer-Drug	Targeting and killing leukemia cells due to enhanced binding affinity and cell internalization via multivalent effects	[114]
	aptNTrs	Targeting human T-cell acute lymphocytic leukemia with high payload of drugs	[115]
Aptamer-conjugated nano-vehicles	DAG-NX213-L	Inhibiting the VEGF-induced endothelial cell proliferation and vascular permeability increase and angiogenesis	[90]
	Aptamosome	Selectively delivering the drug to PSMA-positive prostate cancer cells by Dox-encapsulating liposome conjugated with aptamers	[81]
	TDO5-micelle	Efficient delivering the drug to target cancer cells by aptamer-micelle assembly with high sensitivity and specificity in flow channel system	[118]
	QD-Apt	Delivering Dox to the prostate cancer cells and imaging the cancer cells by quantum dot	[120]
	NP-Apt	Suppressing the metastatic cancer progression and inducing the apoptosis of cancer cells	[79]
	MUC-1 Origami-Dox-AuNRs	Chemotherapeutically and photothermally killing the MUC1-overexpressed multidrug resistant breast cancer cells	[126]

## Abbreviations

MAPK	mitogen-activated protein kinase
PI3K	phosphoinositide 3-kinase
PKC	protein kinase C
STAT	signal transducer and activator of transcription
e-fabp	epidermal fatty acid binding protein
EMT	epithelial-to-mesenchymal transition
